# Supplementary material for: Iterative improvement in the automatic modular design of robot swarms
Source: PeerJ Comput Sci. 2020 Dec 7;6:e322. doi: 10.7717/peerj-cs.322 (PMC7924708; doi:10.7717/peerj-cs.322)
Supplement: Supplemental Information 3 [file peerj-cs-06-322-s003.zip › argos3/doc/api/standalone/a00610_source.html]

ARGoS: plugins/simulator/physics\_engines/dynamics2d/dynamics2d\_model.h Source File


- Main Page
- Related Pages
- Namespaces
- Classes
- Files

- File List
- File Members

# plugins/simulator/physics\_engines/dynamics2d/dynamics2d\_model.h

Go to the documentation of this file.

```
00001 
00007 #ifndef DYNAMICS2D_MODEL_H
00008 #define DYNAMICS2D_MODEL_H
00009 
00010 namespace argos {
00011    class CDynamics2DEngine;
00012 }
00013 
00014 #include <argos3/core/simulator/physics_engine/physics_model.h>
00015 #include <argos3/plugins/simulator/physics_engines/dynamics2d/dynamics2d_engine.h>
00016 
00017 namespace argos {
00018 
00027    class CDynamics2DModel : public CPhysicsModel {
00028 
00029    public:
00030 
00031       typedef std::map<std::string, CDynamics2DModel*> TMap;
00032 
00033    public:
00034 
00035       CDynamics2DModel(CDynamics2DEngine& c_engine,
00036                        CEmbodiedEntity& c_entity) :
00037          CPhysicsModel(c_engine, c_entity),
00038          m_cDyn2DEngine(c_engine) {}
00039 
00040       virtual ~CDynamics2DModel() {}
00041 
00042       virtual void Reset() = 0;
00043 
00048       inline CDynamics2DEngine& GetDynamics2DEngine() {
00049          return m_cDyn2DEngine;
00050       }
00051 
00056       inline const CDynamics2DEngine& GetDynamics2DEngine() const {
00057          return m_cDyn2DEngine;
00058       }
00059 
00060    private:
00061 
00062       CDynamics2DEngine& m_cDyn2DEngine;
00063 
00064    };
00065 
00066 }
00067 
00068 #endif
```

---

Generated on 10 Jul 2018 for ARGoS by 
 1.6.1
